# Nanocell-mediated delivery of miR-34a counteracts temozolomide resistance in glioblastoma

**DOI:** 10.1186/s10020-021-00293-4

**Published:** 2021-03-25

**Authors:** Muhammad Babar Khan, Rosamaria Ruggieri, Eesha Jamil, Nhan L. Tran, Camila Gonzalez, Nancy Mugridge, Steven Gao, Jennifer MacDiarmid, Himanshu Brahmbhatt, Jann N. Sarkaria, John Boockvar, Marc Symons

**Affiliations:** 1 The Elmezzi Graduate School of Molecular Medicine, Northwell Health, Manhasset, NY USA; 2 Karches Center for Oncology, The Institute of Molecular Medicine, The Feinstein Institutes for Medical Research at Northwell Health, 350 Community Drive, Manhasset, NY 11030 USA; 3 Department of Molecular Medicine, Donald and Barbara Zucker School of Medicine at Hofstra/Northwell, Manhasset, NY USA; 4 Department of Cancer Biology, Mayo Clinic Arizona, Scottsdale, AZ USA; 5 EnGeneIC Ltd., Sydney, NSW Australia; 6 Department of Radiation Oncology, Mayo Clinic, Rochester, MN USA; 7 Brain Tumor Center, Lenox Hill Hospital, New York, NY USA; 8 Department of Neurosurgery, Donald and Barbara Zucker School of Medicine at Hofstra/Northwell, Manhasset, NY USA

**Keywords:** Glioblastoma, Temozolomide resistance, MicroRNA delivery, Nanoparticle, Heterogeneity

## Abstract

**Background:**

Glioblastoma is the most common primary brain tumor and remains uniformly fatal, highlighting the dire need for developing effective therapeutics. Significant intra- and inter-tumor heterogeneity and inadequate delivery of therapeutics across blood–brain barrier continue to be significant impediments towards developing therapies which can significantly enhance survival. We hypothesize that microRNAs have the potential to serve as effective therapeutics for glioblastoma as they modulate the activity of multiple signaling pathways, and hence can counteract heterogeneity if successfully delivered.

**Methods:**

Using a computational approach, we identified microRNA-34a as a microRNA that maximally reduces the activation status of the three core signaling networks (the receptor tyrosine kinase, p53 and Rb networks) that have been found to be deregulated in most glioblastoma tumors. Glioblastoma cultures were transfected with microRNA-34a or control microRNA to assess biological function and therapeutic potential in vitro. Nanocells were derived from genetically modified bacteria and loaded with microRNA-34a for intravenous administration to orthotopic patient-derived glioblastoma xenografts in mice.

**Results:**

Overexpression of microRNA-34a strongly reduced the activation status of the three core signaling networks. microRNA-34a transfection also inhibited the survival of multiple established glioblastoma cell lines, as well as primary patient-derived xenograft cultures representing the proneural, mesenchymal and classical subtypes. Transfection of microRNA-34a enhanced temozolomide (TMZ) response in in vitro cultures of glioblastoma cells with primary TMZ sensitivity, primary TMZ resistance and acquired TMZ resistance. Mechanistically, microRNA-34a downregulated multiple therapeutic resistance genes which are associated with worse survival in glioblastoma patients and are enriched in specific tumor spatial compartments. Importantly, intravenous administration of nanocells carrying miR-34a and targeted to epidermal growth factor receptor (EGFR) strongly enhanced TMZ sensitivity in an orthotopic patient-derived xenograft mouse model of glioblastoma.

**Conclusions:**

Targeted bacterially-derived nanocells are an effective vehicle for the delivery of microRNA-34a to glioblastoma tumors. microRNA-34a inhibits survival and strongly sensitizes a wide range of glioblastoma cell cultures to TMZ, suggesting that combination therapy of TMZ with microRNA-34a loaded nanocells may serve as a novel therapeutic approach for the treatment of glioblastoma tumors.

**Supplementary Information:**

The online version contains supplementary material available at 10.1186/s10020-021-00293-4.

## Introduction

Glioblastoma is the most common primary brain tumor. It remains incurable with a bleak prognosis despite aggressive treatment that includes surgical resection and adjuvant combination temozolomide (TMZ) and radiation therapy (Stupp et al. 2005; Hegi et al. 2005). The invasive nature of glioblastoma precludes total surgical resection and resistance to adjuvant combination TMZ and radiation therapy is observed in approximately half of the patients initially diagnosed with glioblastoma and nearly all the patients with recurrent glioblastoma, leading to treatment failure and significant side effects in the few long term survivors (Stupp et al. 2005; Hottinger et al. 2009; Patel et al. 2014; Osuka and Meir 2017; Lee 2016). The results of many clinical trials with targeted therapies have not demonstrated improvement in overall glioblastoma patient survival as evidenced by multiple failed Phase III clinical trials, despite promising results in multiple solid malignancies (Mandel et al. 2018).

Intra-tumoral heterogeneity (ITH)—where cells from the same tumor have distinct phenotypic, genetic and epigenetic states—is well established in glioblastoma at the cellular, temporal and spatial levels (Patel et al. 2014; Parsons et al. 2008; Wang et al. 2016; Jin et al. 2017; Sottoriva et al. 2013). Importantly, sub-clonal populations of tumor cells can have different driver mutations which provide essential growth advantage for neoplastic proliferation and survival and thus such sub-clonal populations in the same tumor exhibit a spectrum of response to both targeted agents and TMZ (Patel et al. 2014; Jin et al. 2017; Sottoriva et al. 2013; Snuderl et al. 2011; Akgul et al. 2019; Segerman et al. 2016; Meyer et al. 2015). Furthermore, increased heterogeneity correlates with worse survival in glioblastoma patients, highlighting the critical role of ITH in therapeutic resistance (Patel et al. 2014). ITH and ineffective drug delivery across the blood–brain and blood-tumor barriers remain as two most significant impediments towards developing therapeutics that can significantly improve clinical outcomes for glioblastoma patients (Patel et al. 2014; Tellingen et al. 2015).

MicroRNAs (miRNA) are small endogenous RNAs that can modulate the expression of multiple targets in the same cell (Rupaimoole and Slack 2017). Therefore, we hypothesized that appropriate miRNAs, once successfully delivered to glioblastoma tumors, can counteract glioblastoma therapeutic resistance resulting from such heterogeneity and optimize treatment, leading to improved patient outcomes. Indeed, a single appropriately selected miRNA may be able to modulate the expression of multiple survival and therapeutic resistance genes and their downstream signaling elements.

To identify candidate therapeutic miRNAs that target all glioblastoma subtypes, we used a bioinformatics approach to search for miRNAs that act on signaling networks that have been shown to be deregulated in glioblastoma. We selected miR-34a-5p (miR-34a) for further investigation and examined its therapeutic effects, both as monotherapy and in combination with TMZ, in a wide range of glioblastoma cultures representing all glioblastoma subtypes, including differentiated and stem-like cultures and cultures with variable TMZ resistance. We show that different therapeutic resistance genes are enriched in unique glioblastoma spatial compartments in the same tumor and that miR-34a can sensitize tumors by down-regulating multiple resistance targets, thus potentially counteracting ITH. Importantly, we effectively delivered miR-34a to orthotopic tumors in vivo, using intravenous administration of the miRNA packaged in bacterially-derived nanocells, targeted to EGFR via a bi-specific single-chain variable fragment (scFv) antibody (Taylor et al. 2015) and showed a marked increase in mouse survival in combination with TMZ treatment.

## Materials and methods

### Cell lines

GBM6, GBM118 and GBM126 are primary patient derived xenografts and were cultured on Laminin (Sigma-Aldrich®) coated flasks in Knockout™ DMEM/F-12 (Gibco™) medium, supplemented with StemPro™ Neural Supplement (Gibco™), recombinant human epidermal growth factor (Gibco™), recombinant human fibroblast growth factor basic (Gibco™) and l-Glutamine (Gibco™). Using RNAseq obtained from orthotopic tumors, GBM6, GBM118 and GBM126 cells were characterized as belonging to classical, proneural and mesenchymal subtypes (PDX National Resource Database: https://www.mayo.edu/research/labs/translational-neuro-oncology/mayo-clinic-brain-tumor-patient-derived-xenograft-national-resource/pdx-phenotype/molecular-subtype). A172, LN229 and T98G were acquired from American Type Culture Collection (ATCC) and cultured in Dulbecco’s Modified Eagle Medium (Gibco™) supplemented with 10% fetal bovine serum (HyClone™) and L-Glutamine (Gibco™). A172 and LN229 cells were grown in culture medium supplemented with IC_50_ dose of TMZ for 2 weeks to derive the TMZ resistant clones: A172TR and LN229TR. All medium was replaced every two days. All cell cultures were confirmed free of mycoplasma contamination.

## Transfections

The following microRNAs were used: miR-34a (sense: rAmCrArAmCmCrArGmCmUrArArGrAmCrAmCmUrGmCmCrA;

antisense: rUrGrGmCrArGrUrGrUrCrUmUrArGrCrUrGrGrUrUrGrU) and control miRNA: C. elegans miR-67 (cel-miR-67) (mature sequence: UCACAACCUCCUAGAAAGAGUAGA). Cel-miR-67 has minimal sequence homology with any mouse, rat or human miRNA and hence is frequently used as a control in miRNA research (Chandrasekaran et al. 2017). These miRNAs were acquired from IDT®. 2-O-methyl modification was added to reduce nuclease degradation and immunostimulation as previously described (Xue et al. 2014). Transfection conditions were optimized, and cells were reverse transfected with 0–30 nM miRNAs in 96 well plates, according to reverse transfection protocol outlined by Dharmacon™. Briefly, 100 µM stock miRNA solution was diluted to the final desired concentration in transfection medium consisting of diluted DharmaFECT 1(Dharmacon™) and Dharmacon™ cell culture reagent (Cat #B-004500–100). This transfection mix was then added to Poly-l-Lysine coated 96 well plates and 30 min later 2000 cells/well were seeded. Transfection with up to 30 nM control did not result in any discernable effects. TOX transfection control (Dharmacon™, Cat#D-001500-01-20) (Orr-Burks et al. 2017) was used to confirm transfection conditions and revealed transfection efficiencies greater than 85% in all cell lines. Three different Bcl2 siRNA were acquired from IDT® in the form of TriFECTa® Kit (design ID: hs.Ri. BCL2.13).

### Cell transductions

Stable cell line with doxycycline inducible expression of miR-34a was generated by utilizing shMIMIC Inducible miR-34a lentivirus (VSH6904-224647620) which was acquired from Dharmacon™ in order to validate the findings from IDT® miR-34a. GBM6 cells cultures were transduced at MOI of 0.9 and efficiently transduced cells were selected with puromycin (1 μg/mL) incubation for three days. For induction, cells were incubated with doxycycline (1 μg/mL) for 72 h, replacing medium with freshly dissolved doxycycline every 24 h.

### Temozolomide treatment and cell proliferation assay

TMZ (Selleckchem, Houston, TX) was added to cells 48 h post transfection with miRNA or 24 h after plating when used as monotherapy. Four days later, cells were fixed with 10% trichloroacetic acid (FisherScientific™) and Sulforhodamine B (SRB) assay was used to quantify cell number (Vichai and Kirtikara 2006).

### Western blot analysis

Glioblastoma cells were transfected with 30 nM miR-34a or control miRNA in 6-well plates (Corning™) and proteins were extracted 48 h post-transfection with M-PER™ (Thermo Scientific™) reagent for in vitro studies with IDT® miR-34a. Proteins were extracted 72 h after doxycycline induction using M-PER™ (Thermo Scientific™) reagent from the GBM6 cell line transduced with lentivirus for stable expression of miR-34a. Proteins were extracted using TRIzol™ (Invitrogen™) reagent for in vivo studies as previously described (Kopec et al. 2017). Bcl2 (2872 s), cMet (8198 s), GAPDH (2118 s), β-tubulin (2146 s), Akt (2920 s), p-Akt (4060 s), Erk (4696 s), and p-Erk (4370 s) antibodies were purchased from Cell Signaling Technologies.

### miRPATH analysis

The reverse search utility of miRPATH v3.0 (Vlachos et al. 2015) was used to identify miRNAs which regulate elements in the glioma pathway (hsa05214) as defined by the Kyoto Encyclopedia of Genes and Genomes (KEGG) (Kanehisa et al. 2016). miRNA-gene interactions from TarBase v 7.0 were used in this analysis. TarBase v 7.0 incorporates more than 600 000 miRNA-gene interactions derived from more than 150 CLIP-seq libraries and hundreds of publications (Vlachos et al. 2014).

### TCGA and Ivy atlas data analysis

The Cancer Genome Atlas (TCGA, HG-UG133A and Agilent-4502A data) and the Ivy atlas data were interrogated through the Gliovis platform (http://gliovis.bioinfo.cnio.es/) (Bowman et al. 2017).

### PCR analysis

Total RNA was isolated using TRIzol™ (Invitrogen™) reagent from snap frozen tumor tissue and cultured cells. The Nanodrop spectrophotometer (Wilmington, DE) was used to assess the purity/concentration of total RNA; RNA samples had 260:280 and 260:230 ratios ≥ 1.9. *MET* (PPH00194A-200) and *RNU*-6 (MS00033740) primers were acquired from Qiagen. *ATM, EGFR, BCL2* and *UGCG* primers were acquired from IDT. Cancer drug resistance PCR array (PAHS-004Z) was acquired from Qiagen and the data was analyzed using The GeneGlobe Data Analysis Center (https://www.qiagen.com/us/shop/genes-and-pathways/data-analysis-center-overview-page). PCR arrays were performed using the Roche 480 Light Cycler instrument, according to the manufacturer’s guidelines. Briefly, qPCR was performed under the following conditions: 95˚C for 10 min, followed by 45 cycles of 95˚C for 15 sec and 60˚C for 1 min. Relative changes in gene expression were calculated as fold-regulation and statistical analyses were done using the web-based portal for RT^2^ Profiler™ PCR Array Data Analysis (Qiagen, Valencia, CA). The cut-off used was fold-regulation difference of 1.5.

### miRNA Nanocell preparation

miR-34a or control miRNA were incubated overnight with nanocells and loading occurred via a method of diffusion. After this, miRNA containing nanocells were incubated with bispecific monoclonal antibodies targeting human EGFR for one day, as previously described in detail (MacDiarmid et al. 2009). To quantify amount of miRNA per nanocell, nanocells were lysed with RLT Buffer (Quiagen), with 20 µl β-ME added to 2 ml RLT. A standard curve was prepared using the quantifluor RNA Kit (Promega) and EDV lysates measured against it using the Quantus fluorometer. Nanocells loaded with miR-34a carried ~ 2200 copies per nanocell.

### Animal studies

Athymic nude mice (nu/nu) were purchased from Charles River. GBM6 cells (500,000) expressing GFP and luciferase were intracranially implanted as previously described (Carlson et al. 2011). In vivo tumor size was quantified by means of quantitative bioluminescent imaging using the IVIS Lumina imaging station (Caliper Life Sciences) (Ozawa and James 2010). Logarithmic tumor growth was confirmed in mice by means of bioluminescence imaging before treatments were started. EGFR-targeted nanocells(10^9^) containing miR-34a or control miRNA (EnGeneIC Pty Ltd, Sydney, Australia) were administered via retro-orbital intravenous injection (Yardeni et al. 2011) on days 31, 33 and 35 post tumor implantation. TMZ was administered (22 mg/kg) via oral gavage for five days from day 32 to 37 post tumor implantation for survival and tumor growth experiments. For mechanistic studies confirming successful delivery of miR-34a by nanocells, 10^9^ nanocells containing miR-34a or control miRNA were administered via intravenous injection on days 31, 32 and 33 and GFP-positive tumors were harvested on day 35 with the aid of a dissecting microscope equipped with epi-fluorescence and immediately snap frozen with liquid nitrogen. Humane treatment of all experimental animals was ensured and all experiments were carried out after approval of the Institutional Animal Care and Use Committee (IACUC).

### Statistical analysis

Unpaired Student’s t-test was used to assess statistical significance between miR-34a-treated and control conditions for in vitro and in vivo studies. Enrichment analysis of miRNA gene targets in KEGG pathways was carried out using the one tailed Fisher’s exact test, often referred to as the hypergeometric test, using miRPATH as previously described (Vlachos et al. 2012). CompuSyn software was used to calculate combination indexes (Chou and Martin 2005) in order to quantify synergistic interaction between miR-34a and TMZ. Log rank test was used to test for significant differences in survival in the in vivo study. Maximally selected rank statistics were used to determine optimal cut-off between high and low expressors of resistance genes for survival analysis. Tukey's Honest Significant Difference test were used to determine significant expression differences between groups in TCGA and Ivy atlas data.

## Results

### miR-34a targets core glioblastoma signaling pathways

In order to identify a suitable miRNA that can counteract both inter- and intra-tumor heterogeneity in glioblastoma amongst miRNAs listed in the Tarbase database (Karagkouni et al. 2017), we utilized miRPATH v 3.0 to conduct a functional meta-analysis of multiple publications and publicly available NGS datasets to search for miRNAs that target the maximum number of oncogene drivers in KEGG glioblastoma driver pathways (see Methods). This analysis revealed that miR-34a regulates the expression of 29 total target genes in glioma signaling networks (p = 1.876079 e-97), which is the largest number of targeted genes of any miRNA listed in the Tarbase database (Additional file 1: Table S1, Additional file 2: Table S2 and Additional file 4: Figure S1). In addition, miR-34a is known to be down-regulated in glioblastoma samples relative to non-neoplastic brain tissue (Rolle 2015; Shea et al. 2016; Gao et al. 2013). Taken together, these findings identify miR-34a as a potential therapeutic miRNA in glioblastoma.

**Fig. 1:**
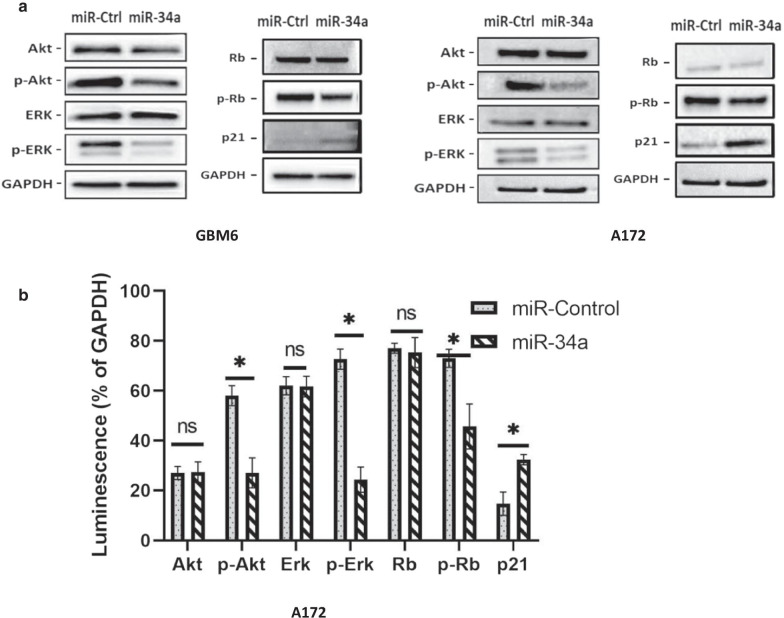
Overexpression of miR-34a decreases oncogenic signaling in glioblastoma cells.** a** Representative western blot images showing reduction in phosphorylated Akt, ERK and Rb and increased p21 expression in miR-34a-transfected relative to control miRNA-transfected GBM6 and A172 glioblastoma cells. Cell lysates were prepared 2 days post-transfection and analyzed by western blotting. **b** Western blot data quantification (% reduction in luminescence relative to GAPDH) in the A172 cell line**.** Histograms showing means (± SD) of luminescence signals from three independent experiments, normalized to that of GAPDH. A paired t-test was used to test for significant difference between mean luminescence values. *p < 0.05

In glioblastoma tumors, different combinations of driver mutations have been shown to deregulate three core signaling networks governed by receptor tyrosine kinases (RTK), p53 and Rb, respectively in both the KEGG and TCGA databases (Verhaak et al. 2010). To determine the effects of miR-34a over-expression on these networks, we examined phosphorylated Akt and ERK as markers for RTK signaling, phosphorylated Rb as a marker for Rb signaling and p21 as a marker for p53 signaling. Transfection with miR-34a caused significant decreases in phosphorylated Akt (50% reduction), ERK (65% reduction) and Rb (40% reduction), along with an increase in the tumor suppressive protein p21 (100% increase) in two different glioblastoma cultures relative to control miRNA transfection (Fig. 1). Thus, overexpression of miR-34a strongly reduces the activation status of the three core signaling networks that are deregulated in glioblastoma.

**Fig. 2:**
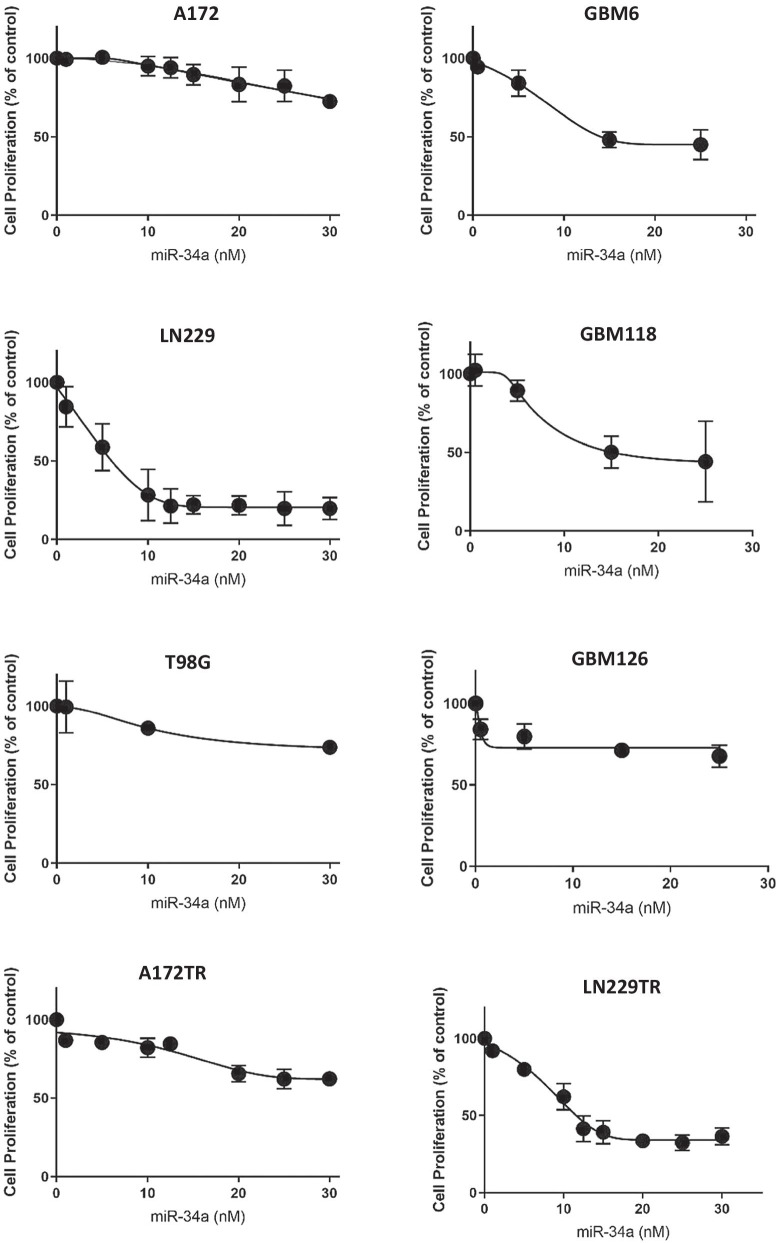
Overexpression of miR-34a inhibits glioblastoma cell proliferation. Glioblastoma cell cultures were reverse-transfected with 0–30 nM miR-34a or 30 nM control miRNA which served as the control condition. Cell proliferation was quantified by SRB assay 4 days post-transfection. All data represent mean SRB values of miR-34a transfected wells as a percentage of control transfected wells (± SD) from three independent experiments

In line with our observation that miR-34a inhibits the function of the RTK, p53 and Rb networks, we found that over-expression of miR-34a inhibits the proliferation of all established glioblastoma cell lines that we have tested, A172, LN229 and T98G, albeit to varying degrees (Fig. 2). To examine whether these results extend to all three subtypes of glioblastoma, we tested a patient-derived primary culture from each glioblastoma subtype, classical (GBM6), mesenchymal (GBM118) and proneural (GBM126). Overexpression of miR-34a resulted in significant inhibition of cell proliferation for all primary cultures (Fig. 2). We also confirmed that the quantitative differences in therapeutic efficacy of miR-34a were not due to variable transfection efficiencies between the respective cell cultures (Additional file 5: Figure S2). Taken together, these data strongly suggest that overexpression of miR-34a can have a therapeutic effect in a wide range of glioblastoma tumors.

### miR-34a enhances temozolomide response in glioblastoma cells

**Fig. 3:**
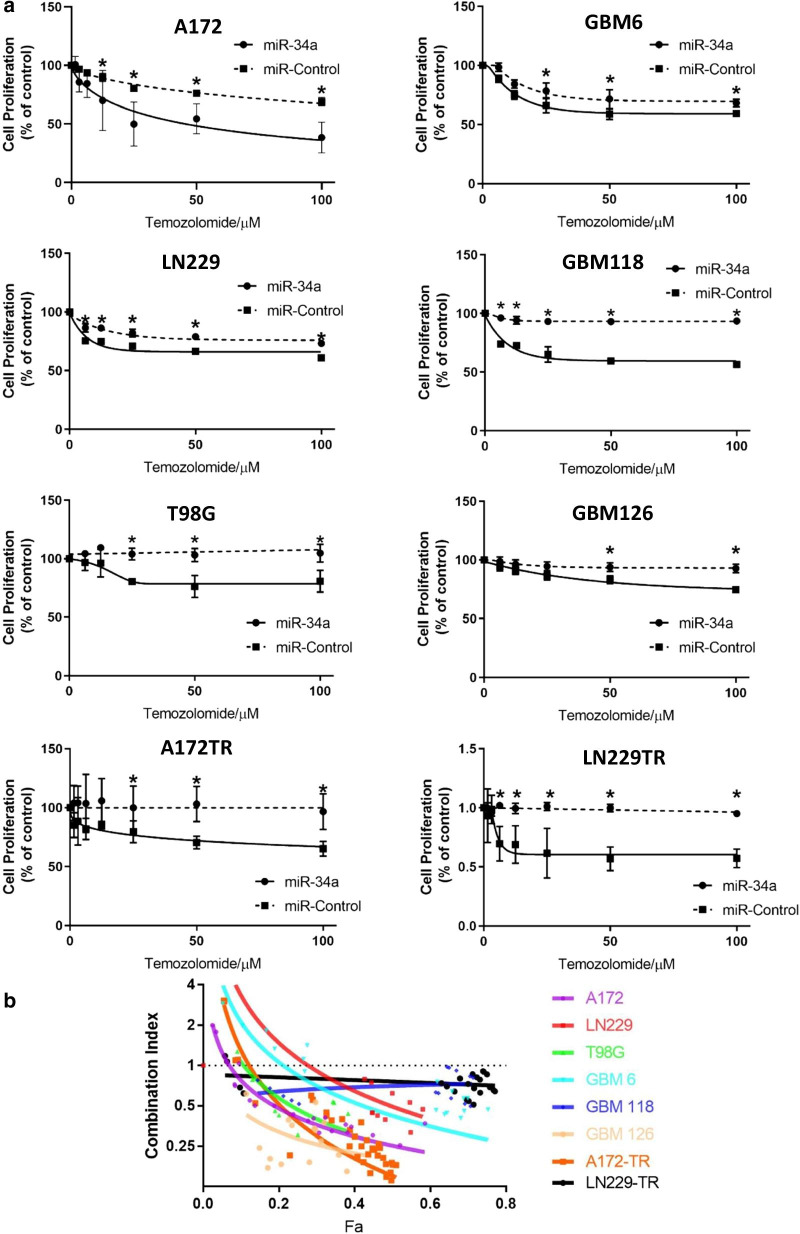
miR-34a sensitizes to TMZ in a wide range of glioblastoma cultures. **a** Glioblastoma cultures were reverse-transfected with miR-34a or control miRNA. Two days later, these cultures were treated with TMZ for 4 days. Dashed lines represent mean SRB values (± SD) of TMZ response in control transfected cells and solid lines represent mean SRB values (± SD) of TMZ response miR-34a transfected cells from three independent experiments. Data are normalized to account for the effect of miR-34a on inhibition of proliferation. A paired t-test was used to test for significant difference between means. *p < 0.05 **b** Glioblastoma cultures were treated as in (**a**) with 0–30 nM miR-34a or control and 0–100 µM TMZ in various combinations and combination index (CI) values were calculated using CompuSyn. Symbols represent actual CI data points and CI trendlines were generated by non-linear regression for A172, LN229, T98G, GBM6, GBM126, A172 TR, LN 229 TR cultures and linear regression for GBM118, LN229 TR cultures (Fa, fraction effected represents % inhibition of proliferation). CI = 1, additivity; CI > 1, antagonism; CI < 1, synergy

As miR-34a targets that are deregulated in glioblastoma have also been implicated in resistance to chemotherapy, we investigated if miR-34a could sensitize glioblastoma cells to TMZ. We first examined the TMZ sensitivity of the three glioblastoma cell lines (A172, LN229 and T98G) and primary cultures (GBM6, GBM118 and GBM126) and observed a range of inhibitory effects on cell survival (Fig. 3). Based on these results, we classified A172, LN229 and GBM6 as primary sensitive, while T98G, GBM118 and GBM126 were classified as primary resistant. Next, we derived A172TR and LN229TR secondary resistant cell lines from the primary sensitive cell lines as an in vitro model of acquired resistance, by prolonged culture of these cells in a low concentration of TMZ (Fig. 3). As shown previously, miR-34a transfection resulted in significant inhibition of proliferation in both secondary resistant cell lines to TMZ as well (Fig. 2).

To examine whether miR-34a sensitizes glioblastoma cells to TMZ, multiple glioblastoma cell cultures were transfected with miR-34a or control miRNA before being treated with TMZ. We observed that miR-34a enhanced TMZ responses in all cell cultures examined, including cells with primary TMZ sensitivity (A172, LN229, GBM6), primary TMZ resistance (T98G, GBM118, GBM126) and secondary acquired TMZ resistance (A172TR, LN229TR) (Fig. 3a). We also determined the combination index (CI) as a quantitative measurement of synergy between two therapeutics (Chou 2010). The CI is essentially a ratio of the effectiveness of two therapeutics in combination to that of those therapeutics used alone to generate the same biological effect. CI values of less than 1 indicate a synergistic effect. Importantly, the interactions between miR-34a and TMZ showed synergism in all cell cultures tested, as demonstrated by combination index analysis (Fig. 3b). Notably, this panel of cell cultures include all three glioblastoma subtypes, cultures with significantly variable baseline TMZ sensitivity and cell cultures with (A172, LN 229) and without (T98G, GBM6, GBM118 and GBM126) methylation of the O^6^-methylguanine-DNA methyltransferase (MGMT) promoter, indicating miR-34a can potentially counteract heterogeneity and treatment resistance. These data illustrate that miR-34a can be a potential treatment modality in patients with primary and importantly in recurrent glioblastoma since most patients with recurrent glioblastoma have TMZ resistance.

### miR-34a sensitizes to temozolomide by downregulating multiple therapeutic resistance proteins

In order to elucidate mechanisms contributing to miR-34a-mediated TMZ sensitization, we used a commercially available PCR array containing 84 bona fide cancer drug resistance genes to identify miR-34a-regulated therapy resistance genes. RT-qPCR was used to confirm successful transfection efficiency and revealed more than 100-fold increase in levels of miR-34a in all cell lines confirming transfection (Additional file 6: Figure S3). We found that miR-34a down-regulated twenty-three resistance genes by more than 35% in at least one of the primary cultures representing proneural, mesenchymal and classical subtypes of glioblastoma. Some of which were shared, while others were unique to each glioblastoma subtype culture (Additional file 3: Table S3).

**Fig. 4:**
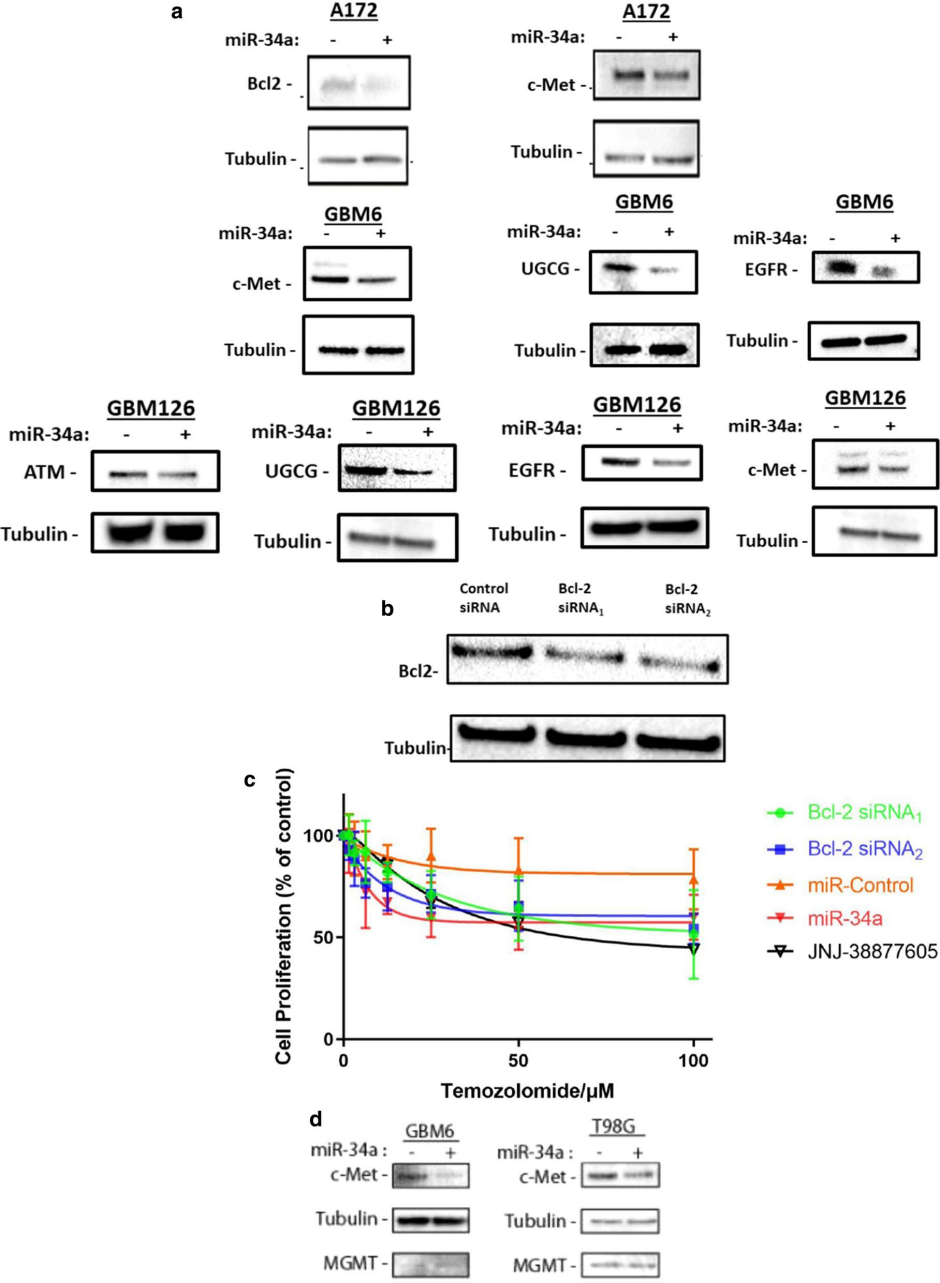
Down-regulation of multiple proteins by miR-34a contributes to sensitization to TMZ. **a** miR-34a reduces protein levels of ATM, Bcl2, EGFR, UGCG and cMET. Representative western blot images show reduction in ATM, Bcl2, EGFR, UGCG and cMET protein levels in glioblastoma cells. Glioblastoma cells were transfected with 30 nM miR-34a or control miRNA, and cells were lysed 48 h later for protein analysis by western blotting. **b** Bcl2 siRNA reduces Bcl2 protein levels. Representative western blot images show reduction in Bcl2 protein levels in A172 glioblastoma cell line. A172 cells were transfected with two different Bcl2 siRNA or control siRNA at a concentration of 30 nM, and cells were lysed 48 h later for protein analysis by western blotting. **c** Inhibition of Bcl2 or cMet sensitizes to temozolomide. A172 cells were reverse-transfected with two different Bcl2 siRNAs, miR-34a and control miRNA 48 h prior to TMZ therapy or treated with JNJ-38877605 2 h prior to being treated with different concentrations of TMZ. Cell proliferation was determined by SRB. All data represent mean SRB values (± SD) from three independent experiments. **d** miR-34a transfection does not alter MGMT levels. Representative western blot images show reduction in cMET, being used as a positive control, while MGMT protein levels are not affected by miR-34a transfection in the T98G and GBM6 glioblastoma cell cultures. T98G cells were transfected and analyzed similarly to A172 cells in (A). GBM6 cells were transduced with a lentivirus, overexpressing miR-34a only upon doxycycline induction. miR-34a expression was induced in GBM6 cultures for 72 h after which cells were lysed for protein extraction

Amongst the genes down-regulated by miR-34a, we selected five genes (*ATM, EGFR, BCL2, MET, UGCG)* for validation as they have been shown pre-clinically to play critical roles in cell survival and therapeutic resistance in the context of glioblastoma (Eich et al. 2013; Munoz et al. 2014; Barvaux et al. 2004; Jun et al. 2007; Giussani et al. 2012). In our analysis of the TCGA database, over-expression of four of these therapeutic resistance genes is also associated with worse patient survival, implicating that their therapeutic knock-down might lead to prolonged survival in treated patients (Additional file 7: Figure S4). We confirmed that transfection with miR-34a resulted in a significant decrease in the protein and mRNA levels of ATM, EGFR, Bcl2, c-Met and UGCG in glioblastoma cells (Fig. 4a and Additional file 6: Figure S3). To further confirm the role of two of these resistance genes in TMZ resistance, we performed experiments to knock-down Bcl2 using two different siRNAs (Fig. 4b) and inhibited cMet using the specific inhibitor JNJ-38877605 (Finisguerra et al. 2015). Both knock-down of Bcl2 and inhibition of cMet strongly sensitized to TMZ therapy (Fig. 4c) indicating that down-regulation of both Bcl2 and cMet likely contribute to TMZ sensitization by miR-34a.

MGMT is a critical contributor to TMZ resistance (Lee 2016). Our search of TarBase v.8—a database which catalogs all the experimentally supported miRNA-gene interactions—revealed no interaction between miR-34a and MGMT. Furthermore, Kupnicka et al. reported no correlation between miR-34a and MGMT levels in 49 glioblastoma patient samples (Jesionek-Kupnicka et al. 2019). In line with these findings, transfection of miR-34a in two different cell lines did not decrease MGMT protein levels (Fig. 4d), suggesting that miR-34a sensitizes to TMZ therapy in a mechanism that is independent of MGMT.

### Significant inter- and intra-tumoral heterogeneity exists in the expression of resistance mechanisms

**Fig. 5:**
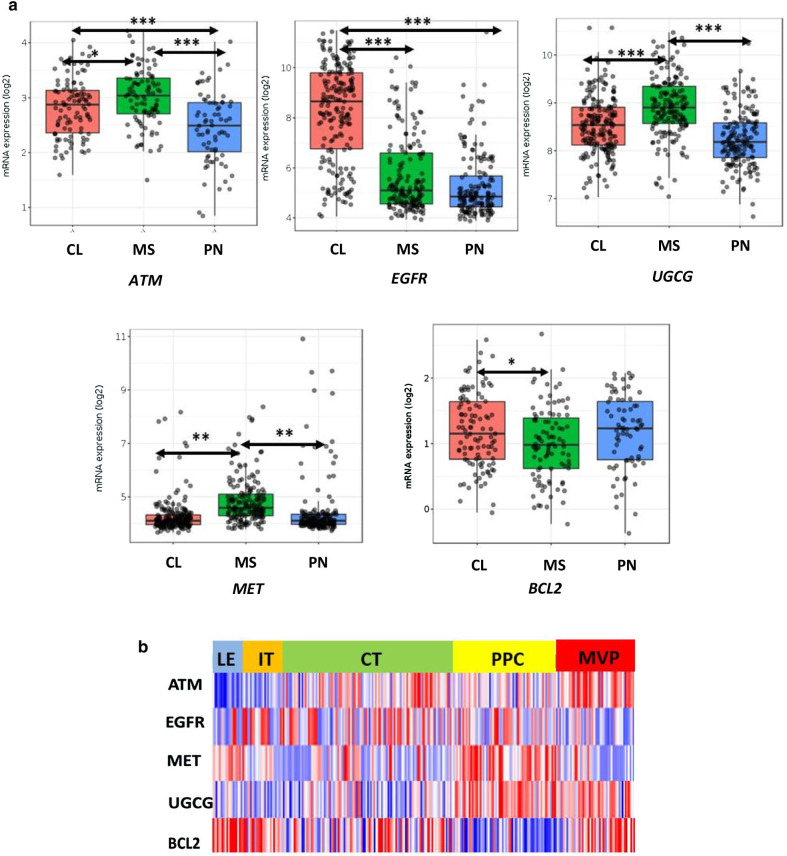
Significant heterogeneity exists in the expression of resistance mechanisms in glioblastoma.** a** Expression of resistance genes in different glioblastoma subtypes. Log2 mRNA expression of resistance genes in classical (orange), mesenchymal (green) and proneural (blue) subtypes. Tukey HSD test was used to assess statistical significance. ***p < 0.001; **p < 0.01; *p < 0.05. Horizontal lines refer to the groups being compared for statistical analysis. CL: classical glioblastoma subtype, MS: mesenchymal glioblastoma subtype, PN: proneural glioblastoma subtype. **b** Different therapeutic resistance mechanisms are enriched in distinct spatial compartments of glioblastoma. Heatmap of relative mRNA expression of resistance genes from different tumor areas. LE: leading edge, IT: infiltrating tumor, CT: cellular tumor, PPC: pseudo-palisading cells, MVP: microvascular proliferation

Our PCR and western blot experiments revealed that some therapeutic resistance genes may be differentially expressed between the proneural, mesenchymal and classical primary cultures that we have examined (Additional file 8: Figure S5). To determine the validity and generalizability of these findings, we interrogated the Ivy GAP database and examined the expression of these resistance genes in different glioblastoma subtypes. In congruence with our in vitro data, the expression of this set of resistance genes was significantly different amongst subtypes of glioblastoma (Fig. 5a). These data suggest that different subtypes might rely on distinct therapeutic resistance mechanisms, highlighting the presence of inter-tumoral heterogeneity.

Next, we asked if the resistance genes that we identified also exhibit intra-tumoral heterogeneity. Interestingly, our analysis of the Ivy GAP atlas revealed that there is significant enrichment of resistance genes in distinct spatial regions within the same tumor. For example, *ATM* is highly expressed in the microvascular proliferative zones of the tumor, *EGFR* is highly expressed in the cellular bulk of the tumor while *MET* is enriched in the leading edges and the necrotic tumor regions. Importantly, miR-34a-modulated therapeutic resistance genes were expressed in all different spatial compartments, (Fig. 5b). Enrichment of resistance genes in the respective spatial compartments suggests that glioblastoma tumors rely on distinct mechanisms to resist therapeutic efforts in different parts of the tumor and that their simultaneous targeting will be essential to achieve an optimal therapeutic response. Importantly however, miR-34a targets multiple resistance genes, such that it down-regulates at least one enriched gene in each tumor compartment and thus can potentially counteract heterogeneity arising due to spatial heterogeneity of resistance gene expression to sensitize the entire tumor.

### Nanocells packaged with miR-34a sensitize to TMZ in orthotopic glioblastoma tumors

**Fig. 6:**
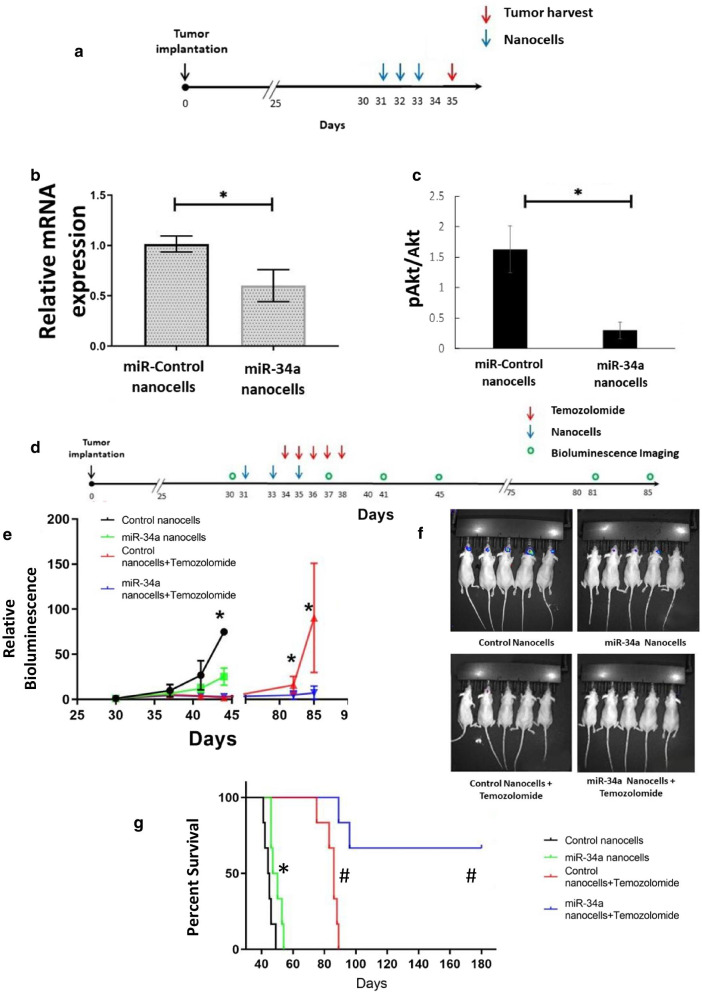
In vivo delivery of miR-34a via nanocells sensitizes to TMZ. **a**–**c** Intravenously-administered nanocells deliver miR-34a to orthotopically implanted tumors. Five hundred thousand GBM6 cells expressing firefly luciferase were implanted in the striatum of female athymic nude mice. Six mice were used in each group. Three doses of 10^9^ nanocells packaging either miR-34a or control miRNA were injected intravenously on days 30, 31 and 32 post-tumor implantation and tumors were harvested day 35 post implantation for analysis of cMet mRNA expression by RT-qPCR (**b**) and phospho-Akt levels by western blotting (**c**). Data represent mean relative expression (± SD); a paired t-test was used to test for significant difference between means, *p < 0.05. **d**–**g** miR-34a inhibits tumor growth, improves survival and sensitizes to TMZ. Six mice were used in each experimental group. Tumor cell implantation was carried out as described under (**a**–**c**). Three doses of 10^9^ nanocells packaging either miR-34a or control miRNA were injected intravenously on days 31, 33 and 35 and TMZ was administered via oral gavage on days 34, 35, 36, 37 and 38 post-tumor implantation. (**e**) Tumor growth was monitored by bioluminescence imaging. Data represents mean total flux (± SD) and a paired t-test was used to test for significant difference between means; *p < 0.05. (**f**) Representative bioluminescent imaging from Day 43 post tumor implantation. (**g**) Survival of mice shown as Kaplan–Meier survival curves. Log rank test was used to assess statistical significance, (n = 6); *p < 0.05 and #p < 0.001 vs control nanocell-treated mice

Bacterially-derived nanocells (EnGeneIC Pty Ltd) (MacDiarmid et al. 2009, 2007) have been shown to deliver doxorubicin to glioblastoma tumors (MacDiarmid et al. 2016) and miRNA to mesothelioma (Reid et al. 2013) in vivo. For these experiments, the nanocells were loaded with miR-34a or control miRNA and with a bispecific antibody against EGFR on glioma tumor cells. To determine if nanocells can deliver therapeutic amounts of miR-34a to orthotopic implanted glioblastoma tumors, nanocells were injected intravenously and levels of cMet mRNA and phospho-Akt—which we showed are reduced by successful miR-34a transfection in vitro—were used to assess in vivo miR-34a delivery by nanocells. We observed that intravenous administration of miR-34a nanocells reduced both cMet and phosphorylated Akt levels in orthotopically implanted glioblastoma tumors to a similar extent as in vitro transfection (Fig. 6a–c).

To determine whether miR-34a also enhances the therapeutic effect of TMZ in vivo, mice implanted with orthotopic glioblastoma tumors were treated with combination of intravenously injected nanocells and TMZ administered by oral gavage (Fig. 6d). Administration of miR-34a nanocells resulted in a modest (75%), but statistically significant reduction in tumor growth, as determined by bioluminescence imaging, and increased animal survival by a median of four days (Fig. 6e, f). Strikingly, whereas TMZ monotherapy for five days caused a median increase of 40 days in mouse survival, combination therapy with miR-34a nanocells resulted in long-term survival of most of the treated mice (Fig. 6f). The survival study was terminated 180 days post-tumor implantation. At this point, the only remaining mice were from the TMZ and miR-34a combination group and none had any evidence of tumor on bioluminescence imaging or visual inspection of harvested mouse brains. Thus, these results show that intravenously administered nanocells can deliver therapeutic amounts of miR-34a to orthotopic glioblastoma tumors and sensitize to TMZ therapy.

## Discussion

In this paper, we identify miR-34a as a microRNA that inhibits the proliferation of a wide spectrum of glioblastoma cells, including primary patient-derived cultures belonging to the three subtypes of glioblastoma. We demonstrate that overexpression of miR-34a strongly sensitizes both primary and established glioblastoma cultures to TMZ, including cells that show either primary or secondary resistance to TMZ. We show that the expression of therapeutic resistance genes is subtype-specific and spatially heterogeneous, which strongly suggests that their simultaneous targeting will be necessary to achieve therapeutic benefit. This can potentially be achieved by therapeutic delivery of miR-34a as it down-regulates multiple therapeutic resistance genes in all spatial compartments of glioblastoma. Importantly, we also provide evidence that miR-34a can effectively be delivered to an orthotopic patient-derived mouse xenograft using nanocells, leading to long term survival when treated in combination with TMZ. These results suggest that delivery of miR-34a may be a powerful new adjuvant for the treatment of glioblastoma in combination with TMZ that can mitigate inter- and intra-tumor heterogeneity.

TMZ resistance remains a critical challenge in the clinical care of patients afflicted with glioblastoma. The remarkable result that miR-34a transfection sensitizes to TMZ all cell lines and primary patient-derived cultures that we have examined, suggests that delivery of miR-34a may be a powerful new adjuvant for the treatment of glioblastoma in combination with TMZ. Notably, the cell cultures that we have used represent a wide spectrum of genomic backgrounds representing all molecular subtypes of glioblastoma, variable phenotypes and sensitivity to TMZ and RT and includes cultures with and without MGMT promoter-methylation. Targeting multiple resistance pathways is crucial because glioblastoma can harbor multiple resistance mechanisms as shown by our data, and due to intra-tumoral heterogeneity, it is likely that resistant nests of cells will survive the initial treatment and lead to recurrence. Our findings suggest that miR-34a can potentially counteract therapeutic resistance resulting from such heterogeneity in glioblastoma.

miR-34a is thought to function as a tumor suppressor miRNA, both in glioblastoma and other cancers (Hermeking 2010; Farooqi et al. 2017; Zhang et al. 2019). In support of this, our results show that transfection of miR-34a decreases levels of phosphorylated Akt, Erk and Rb, and increases expression of p21 in glioblastoma, all of which are critical oncogenic signaling networks in glioblastoma (Verhaak et al. 2010). Additionally, we show that miR-34a down-regulates levels of multiple proteins which are known to contribute to therapeutic resistance. Thus, miR-34a gains its remarkable sensitization modality by targeting multiple pathways and that this results in a reduced likelihood of therapeutic resistance. We therefore expect that delivery of miR-34a should enhance the therapeutic benefits of TMZ treatment in patients with TMZ-resistant tumors and potentially may even allow for lowering the TMZ dose in patients with TMZ-sensitive tumors, thereby decreasing side effects.

Delivery of miRNA remains a substantial hurdle in developing miRNA therapeutics for glioblastoma. Different formulations of synthetic and natural nanocarriers have been reported to successfully package miRNA with robust anti-tumor activity in vitro. These include bio-reducible polymeric nanoparticles (Lopez-Bertoni et al. 2018), PEG-PLGA nanoparticles (Malhotra et al. 2018), functionalized nanogels (Shatsberg et al. 2016), poly-glycerol based polyplexes (Ofek et al. 2016) and exosomes (Katakowski et al. 2013). However, the in vivo efficacy of these delivery platforms was demonstrated in subcutaneous tumors which lack the blood brain barrier, or they were delivered by repeated intra-tumoral injections, which is not feasible for glioma patients. An exception is the three-way-junction-based RNA nanoparticles (3WJmiR-21), which was recently used to deliver an antagomiR of oncogenic miR-21 to orthotopically implanted tumors with intravenous administration, albeit with very modest therapeutic efficacy compared to nanocell mediated delivery of miR-34a (Lee et al. 2017).

Nanocells have been shown to be highly effective in the delivery of both chemotherapy and miRNA to a range of tumors (Taylor et al. 2015; MacDiarmid and Brahmbhatt 2011). The in vivo miR-34a delivery by nanocells surpassed our expectations because of the large size of nanocells. Nanocells are 400 ± 20 nm in diameter, and although nanoparticles with a diameter of up to 800 nm are known to cross the blood brain barrier (BBB) and accumulate in brain parenchyma (MacDiarmid and Brahmbhatt 2011; Betzer et al. 2017), nanoparticles must be small (< 200 nm) for them to effectively penetrate and diffuse through the brain parenchyma (Thorne and Nicholson 2006; Nance et al. 2012). Nanocells successfully delivered miR-34a to the tumor as evidenced by the strong reduction of cMet by RT-qPCR and phospho-Akt by western blotting. Nanocells are known to initially accumulate in the tumor by the enhanced permeability and retention (EPR) effect, which is active in most solid cancers (MacDiarmid and Brahmbhatt 2011). Further, the BBB is known to be locally disrupted in the glioblastoma core (Wolburg et al. 2012) and the LPS (lipopolysaccharide) moiety on the surface of our nanocells may have stimulated a local inflammatory reaction and disrupted the BBB (Banks et al. 2015), with the potential to further enhance nanocell delivery into the tumor. Thus, we hypothesize that the nanocells are preferentially taken up by glioblastoma cells located in the perivascular area, where they accumulate by a combination of the EPR effect and localized disruption of the BBB. It is possible that these initially transfected cells subsequently package miR-34a into extracellular vesicles (EV), leading to paracrine transfer of miR-34a to the remainder of the tumor (Fareh et al. 2017; Zhang et al. 2015). Furthermore, a positive feedback loop such that exogenously transfected miR-34a induces expression of endogenous miR-34a has been described in the literature (Okada et al. 2014; Navarro and Lieberman 2015). This might be another contributing factor to the remarkable delivery of miR-34a by nanocells. Future experiments will explore the mechanistic basis for the efficacy of nanocell-mediated delivery of miR-34a in detail. It is noteworthy that no toxicity of nanocells loaded with doxorubicin was observed in previous studies using canine models of glioblastoma or nanocells loaded with paclitaxel in phase I clinical trials advanced solid malignancies (MacDiarmid et al. 2016; Solomon et al. 2015). We also note that nanocells have recently been shown to induce both innate and other adaptive antitumor immune responses (Sagnella et al. 2020) and it therefore would be of great interest to examine the effect of miR-34a in immunocompetent models.

## Conclusions

Glioblastoma remains uniformly fatal, despite intensive therapy, in large part due to the resistance of these tumors to both radio- and chemo-therapy. miR-34a is a therapeutic miRNA that inhibits the survival of a wide range of glioblastoma cell cultures and strongly enhances TMZ response, even in glioblastoma cells that are highly TMZ-resistant. Glioblastoma tumors rely on multiple resistance mechanisms implying that their simultaneous targeting will be essential to achieving an optimal therapeutic response. Importantly, we show that intravenous delivery of miR-34a, packaged in bacterially-derived nanocells, strongly enhances the therapeutic effects of TMZ in an orthotopic mouse model of glioblastoma. Taken together, our results suggest that targeted nanocell-mediated delivery of miR-34a may be a powerful new adjuvant for the treatment of glioblastoma in combination with TMZ that can mitigate both inter- and intra-tumor heterogeneity and provide a preclinical basis for further evaluation of miR-34a nanocell therapy in human clinical trials.

## Supplementary Information

**Additional file 1: Table S1.** List of genes predicted to be regulated by miR-34a in glioblastoma driver pathways. miRPATH v.3 was used to identify genes in the KEGG glioma pathway which are predicted to be regulated by miR-34a. miRNA-gene interactions cataloged in Tarbase v.7 were used. The cell lines, tissues of origin and the methods used to identify miR-34a gene interactions are included. IP, immunoprecipitation, RA, Reporter Gene assay, WB, Western Blot, MA, microarrays, Bi, Biotin, qP, quantitative polymerase chain reaction, CLASH, crosslinking, ligation, and sequencing of hybrids.

**Additional file 2: Table S2.** List of miRNAs which regulate glioblastoma driver pathways**.** miRPATH v 3.0 was used to explore miRNAs which regulate driver pathways in glioma as described in KEGG database. miRNA–gene interactions cataloged in Tarbase v.7 were used in this search. miRPATH v 3.0 was used to calculate enrichment p values by the one tailed Fisher’s exact test. Top thirty miRNAs from this search are listed.

**Additional file 3: Table S3.** miR-34a down-regulates multiple therapeutic resistance genes. GBM6, GBM118 and GBM126 primary cultures were transfected with 30 nM miR-34a or 30 nM miR-C. Total RNA was extracted 48 h post transfection and RT^2^ Profiler™ PCR Array from Qiagen was used to examine expression changes of 84 drug resistance genes. The table shows fold-regulation of genes with fold-regulation cut-off > 1.5. Fold-change was calculated by dividing the normalized gene expression (2^ (- Delta CT)) in the miR-34a transfected cells by normalized gene expression (2^ (- Delta CT)) in the miR-C transfected cells. Fold-Regulation is equal to fold-change for fold-change values > 1. For fold-change values < 1, fold-regulation is the negative inverse of fold-change.

**Additional file 4: Figure S1.** miR-34a modulates the expression of multiple signaling elements in glioma. The glioma pathway from KEGG (hsa05214) with miR-34a targets is illustrated. Yellow boxes represent genes whose expression has been reported to be modulated by miR-34a in the TarBase v 7.0. Yellow boxes with red outlines are down-regulated, while yellow boxes with blue outlines are up-regulated by miR-34a. Green boxes represent glioma signaling elements not modulated by miR-34a.

**Additional file 5: Figure S2.** Transfection efficiency is comparable across the different cell lines tested. Cells were reverse-transfected with the TOX transfection siRNA. Successful transfection results in cell death which was quantified by SRB assay. All data represent mean SRB values (± SD) from three independent experiments.

**Additional file 6: Figure S3.** miR-34a downregulates multiple therapeutic resistance genes. A. Transfection with miR-34a significantly increases miR-34a expression in glioblastoma primary cultures**.** GBM6, GBM118 and GBM126 cells were transfected with 30 nM control miRNA or miR-34a. Total RNA was isolated 48 h post transfection. RNU-6 was used as housekeeping gene and relative expression calculations were made according to the Livak method. Data show mean ± SD of three technical replicates. B. miR-34a reduces to expression of multiple therapeutic resistance genes in glioblastoma. RTqPCR experiments were performed with independent primers to verify the results of the PCR array. In some instances, expression of genes could not be detected in one or more cultures by the PCR. The corresponding bars are left missing from the figure. RNU-6 was used as housekeeping gene and relative expression calculations were made according to the Livak method. Data was normalized to non-transfected controls and show mean ± SD of three technical replicates. *p < 0.05 of miR-34a cells compared to control transfected cells, n/s implies p > 0.05

**Additional file 7: Figure S4.** High expression of miR-34a down-regulated therapeutic resistance genes is associated with worse survival. Kaplan-Meir curves for overall survival in 585 glioblastoma patients from TCGA dataset for the validated resistance genes. High expressors are plotted in orange and low expressors in blue. Cut-off mRNA expression scores used included ATM (5.47), EGFR (6.6)MET (4.37), BCL2 (3.9) and UGCG (7.8). Maximally selected rank statistic was used to stratify patients into high and low expressors. The log rank test was used to assess statistical significance.

**Additional file 8: Figure S5.** Glioblastoma cultures have different baseline expression of therapeutic resistance gens. Total RNA was extracted from GBM6, GBM118 and GBM126 primary cultures and RT^2^ Profiler™ PCR Array from Qiagen was used to determine baseline levels of *ATM, BCL2, EGFR, MET and UGCG* in these cultures. To determine relative mRNA expression, fold-change was calculated by dividing the normalized gene expression (2^ (- Delta CT)) in the GBM6 and GBM126 cells by normalized gene expression (2^ (- Delta CT)) in the GBM118 cells.

## Data Availability

All datasets used and analyzed in this manuscript are publicly available. See methods for access details.

## Abbreviations

BBB: Blood–brain barrier
CI: Combination index
EGFR: Epidermal growth factor receptor.
EPR: Enhanced permeability and retention
EV: Extracellular vesicles
ITH: Intra and inter-tumor heterogeneity
KEGG: Kyoto Encyclopedia of Genes and Genomes
LPS: Lipopolysaccharide
miRNA: MicroRNA
miR-34a: MicroRNA-34a-5p
RTK: Receptor tyrosine kinases
scFv: Single-chain variable fragment
SRB: Sulforhodamine B
TCGA: The Cancer Genome Atlas
TMZ: Temozolomide

## References

[CR1] Akgul S, Patch AM, D'Souza RCJ, Mukhopadhyay P, Nones K, Kempe S (2019). Intratumoural heterogeneity underlies distinct therapy responses and treatment resistance in glioblastoma. Cancers (Basel)..

[CR2] Banks WA, Gray AM, Erickson MA, Salameh TS, Damodarasamy M, Sheibani N (2015). Lipopolysaccharide-induced blood-brain barrier disruption: roles of cyclooxygenase, oxidative stress, neuroinflammation, and elements of the neurovascular unit. J Neuroinflamm..

[CR3] Barvaux VA, Lorigan P, Ranson M, Gillum AM, McElhinney RS, McMurry TB (2004). Sensitization of a human ovarian cancer cell line to temozolomide by simultaneous attenuation of the Bcl-2 antiapoptotic protein and DNA repair by O6-alkylguanine-DNA alkyltransferase. Mol Cancer Ther.

[CR4] Betzer O, Shilo M, Opochinsky R, Barnoy E, Motiei M, Okun E (2017). The effect of nanoparticle size on the ability to cross the blood-brain barrier: an in vivo study. Nanomedicine (Lond).

[CR5] Bowman RL, Wang Q, Carro A, Verhaak RG, Squatrito M (2017). GlioVis data portal for visualization and analysis of brain tumor expression datasets. Neuro Oncol..

[CR6] Carlson BL, Pokorny JL, Schroeder MA, Sarkaria JN (2011). Establishment, maintenance and in vitro and in vivo applications of primary human glioblastoma multiforme (GBM) xenograft models for translational biology studies and drug discovery. Curr Protoc Pharmacol..

[CR7] Chandrasekaran KS, Sathyanarayanan A, Karunagaran D (2017). miR-214 activates TP53 but suppresses the expression of RELA, CTNNB1, and STAT3 in human cervical and colorectal cancer cells. Cell Biochem Funct.

[CR8] Chou TC (2010). Drug combination studies and their synergy quantification using the Chou-Talalay method. Cancer Res.

[CR9] Chou TC, Martin N (2005). CompuSyn for drug combinations: PC software and user’s guide: a computer program for quantitation of synergism and antagonism in drug combinations, and the determination of IC50 and ED50 and LD50 values.

[CR10] Eich M, Roos WP, Nikolova T, Kaina B (2013). Contribution of ATM and ATR to the resistance of glioblastoma and malignant melanoma cells to the methylating anticancer drug temozolomide. Mol Cancer Ther.

[CR11] Fareh M, Almairac F, Turchi L, Burel-Vandenbos F, Paquis P, Fontaine D (2017). Cell-based therapy using miR-302-367 expressing cells represses glioblastoma growth. Cell Death Dis..

[CR12] Farooqi AA, Tabassum S, Ahmad A (2017). MicroRNA-34a: a versatile regulator of myriads of targets in different cancers. Int J Mol Sci..

[CR13] Finisguerra V, Di Conza G, Di Matteo M, Serneels J, Costa S, Thompson AA (2015). MET is required for the recruitment of anti-tumoural neutrophils. Nature.

[CR14] Gao H, Zhao H, Xiang W (2013). Expression level of human miR-34a correlates with glioma grade and prognosis. J Neurooncol.

[CR15] Giussani P, Bassi R, Anelli V, Brioschi L, De Zen F, Riccitelli E (2012). Glucosylceramide synthase protects glioblastoma cells against autophagic and apoptotic death induced by temozolomide and Paclitaxel. Cancer Invest.

[CR16] Hegi ME, Diserens A-C, Gorlia T, Hamou M-F, de Tribolet N, Weller M (2005). MGMT gene silencing and benefit from temozolomide in glioblastoma. N Engl J Med.

[CR17] Hermeking H (2010). The miR-34 family in cancer and apoptosis. Cell Death Differ.

[CR18] Hottinger AF, Yoon H, DeAngelis LM, Abrey LE (2009). Neurological outcome of long-term glioblastoma survivors. J Neurooncol.

[CR19] Jesionek-Kupnicka D, Braun M, Trabska-Kluch B, Czech J, Szybka M, Szymanska B (2019). MiR-21, miR-34a, miR-125b, miR-181d and miR-648 levels inversely correlate with MGMT and TP53 expression in primary glioblastoma patients. Arch Med Sci AMS.

[CR20] Jin X, Kim LJY, Wu Q, Wallace LC, Prager BC, Sanvoranart T (2017). Targeting glioma stem cells through combined BMI1 and EZH2 inhibition. Nat Med.

[CR21] Jun HT, Sun J, Rex K, Radinsky R, Kendall R, Coxon A (2007). AMG 102, a fully human anti-hepatocyte growth factor/scatter factor neutralizing antibody, enhances the efficacy of temozolomide or docetaxel in U-87 MG cells and xenografts. Clin Cancer Res.

[CR22] Kanehisa M, Furumichi M, Tanabe M, Sato Y, Morishima K (2016). KEGG: new perspectives on genomes, pathways, diseases and drugs. Nucleic Acids Res.

[CR23] Karagkouni D, Paraskevopoulou MD, Chatzopoulos S, Vlachos IS, Tastsoglou S, Kanellos I (2017). DIANA-TarBase v8: a decade-long collection of experimentally supported miRNA–gene interactions. Nucleic Acids Res.

[CR24] Katakowski M, Buller B, Zheng X, Lu Y, Rogers T, Osobamiro O (2013). Exosomes from marrow stromal cells expressing miR-146b inhibit glioma growth. Cancer Lett.

[CR25] Kopec AM, Rivera PD, Lacagnina MJ, Hanamsagar R, Bilbo SD (2017). Optimized solubilization of TRIzol-precipitated protein permits Western blotting analysis to maximize data available from brain tissue. J Neurosci Methods.

[CR26] Lee SY (2016). Temozolomide resistance in glioblastoma multiforme. Genes Dis.

[CR27] Lee TJ, Yoo JY, Shu D, Li H, Zhang J, Yu JG (2017). RNA nanoparticle-based targeted therapy for glioblastoma through inhibition of oncogenic miR-21. Mol Ther.

[CR28] Lopez-Bertoni H, Kozielski KL, Rui Y, Lal B, Vaughan H, Wilson DR (2018). Bioreducible polymeric nanoparticles containing multiplexed cancer stem cell regulating mirnas inhibit glioblastoma growth and prolong survival. Nano Lett.

[CR29] MacDiarmid JA, Brahmbhatt H (2011). Minicells: versatile vectors for targeted drug or si/shRNA cancer therapy. Curr Opin Biotechnol.

[CR30] MacDiarmid JA, Mugridge NB, Weiss JC, Phillips L, Burn AL, Paulin RP (2007). Bacterially derived 400 nm particles for encapsulation and cancer cell targeting of chemotherapeutics. Cancer Cell.

[CR31] MacDiarmid JA, Amaro-Mugridge NB, Madrid-Weiss J, Sedliarou I, Wetzel S, Kochar K (2009). Sequential treatment of drug-resistant tumors with targeted minicells containing siRNA or a cytotoxic drug. Nat Biotechnol.

[CR32] MacDiarmid JA, Langova V, Bailey D, Pattison ST, Pattison SL, Christensen N (2016). Targeted doxorubicin delivery to brain tumors via minicells: proof of principle using dogs with spontaneously occurring tumors as a model. PLoS ONE.

[CR33] Malhotra M, Sekar TV, Ananta JS, Devulapally R, Afjei R, Babikir HA (2018). Targeted nanoparticle delivery of therapeutic antisense microRNAs presensitizes glioblastoma cells to lower effective doses of temozolomide in vitro and in a mouse model. Oncotarget.

[CR34] Mandel JJ, Yust-Katz S, Patel AJ, Cachia D, Liu D, Park M (2018). Inability of positive phase II clinical trials of investigational treatments to subsequently predict positive phase III clinical trials in glioblastoma. Neuro Oncol.

[CR35] Meyer M, Reimand J, Lan X, Head R, Zhu X, Kushida M (2015). Single cell-derived clonal analysis of human glioblastoma links functional and genomic heterogeneity. Proc Natl Acad Sci U S A.

[CR36] Munoz J, Rodriguez-Cruz V, Greco S, Ramkissoon S, Ligon K, Rameshwar P (2014). Temozolomide resistance in glioblastoma cells occurs partly through epidermal growth factor receptor-mediated induction of connexin 43. Cell Death Dis.

[CR37] Nance EA, Woodworth GF, Sailor KA, Shih T-Y, Xu Q, Swaminathan G (2012). A dense poly(ethylene glycol) coating improves penetration of large polymeric nanoparticles within brain tissue. Sci Transl Med..

[CR38] Navarro F, Lieberman J (2015). miR-34 and p53: new insights into a complex functional relationship. PLoS ONE.

[CR39] Ofek P, Calderon M, Mehrabadi FS, Krivitsky A, Ferber S, Tiram G (2016). Restoring the oncosuppressor activity of microRNA-34a in glioblastoma using a polyglycerol-based polyplex. Nanomed Nanotechnol Biol Med.

[CR40] Okada N, Lin C-P, Ribeiro MC, Biton A, Lai G, He X (2014). A positive feedback between p53 and miR-34 miRNAs mediates tumor suppression. Genes Dev.

[CR41] Orr-Burks NL, Shim B-S, Wu W, Bakre AA, Karpilow J, Tripp RA (2017). MicroRNA screening identifies miR-134 as a regulator of poliovirus and enterovirus 71 infection. Sci Data.

[CR42] Osuka S, Van Meir EG (2017). Overcoming therapeutic resistance in glioblastoma: the way forward. J Clin Invest.

[CR43] Ozawa T, James CD (2010). Establishing intracranial brain tumor xenografts with subsequent analysis of tumor growth and response to therapy using bioluminescence imaging. J Vis Exp JoVE.

[CR44] Parsons DW, Jones S, Zhang X, Lin JC, Leary RJ, Angenendt P (2008). An integrated genomic analysis of human glioblastoma multiforme. Science (New York, NY).

[CR45] Patel AP, Tirosh I, Trombetta JJ, Shalek AK, Gillespie SM, Wakimoto H (2014). Single-cell RNA-seq highlights intratumoral heterogeneity in primary glioblastoma. Science.

[CR46] Reid G, Pel ME, Kirschner MB, Cheng YY, Mugridge N, Weiss J (2013). Restoring expression of miR-16: a novel approach to therapy for malignant pleural mesothelioma. Ann Oncol.

[CR47] Rolle K (2015). miRNA Multiplayers in glioma. From bench to bedside. Acta Biochim Polonica..

[CR48] Rupaimoole R, Slack FJ (2017). MicroRNA therapeutics: towards a new era for the management of cancer and other diseases. Nat Rev Drug Discov.

[CR49] Sagnella SM, Yang L, Stubbs GE, Boslem E, Martino-Echarri E, Smolarczyk K (2020). Cyto-immuno-therapy for cancer: a pathway elicited by tumor-targeted, cytotoxic drug-packaged bacterially derived nanocells. Cancer Cell.

[CR50] Segerman A, Niklasson M, Haglund C, Bergstrom T, Jarvius M, Xie Y (2016). Clonal variation in drug and radiation response among glioma-initiating cells is linked to proneural-mesenchymal transition. Cell Rep.

[CR51] Shatsberg Z, Zhang X, Ofek P, Malhotra S, Krivitsky A, Scomparin A (2016). Functionalized nanogels carrying an anticancer microRNA for glioblastoma therapy. J Control Release.

[CR52] Shea A, Harish V, Afzal Z, Chijioke J, Kedir H, Dusmatova S (2016). MicroRNAs in glioblastoma multiforme pathogenesis and therapeutics. Cancer Med.

[CR53] Snuderl M, Fazlollahi L, Le LP, Nitta M, Zhelyazkova BH, Davidson CJ (2011). Mosaic amplification of multiple receptor tyrosine kinase genes in glioblastoma. Cancer Cell.

[CR54] Solomon BJ, Desai J, Rosenthal M, McArthur GA, Pattison ST, Pattison SL (2015). A first-time-in-human phase I clinical trial of bispecific antibody-targeted, paclitaxel-packaged bacterial minicells. PLoS ONE.

[CR55] Sottoriva A, Spiteri I, Piccirillo SG, Touloumis A, Collins VP, Marioni JC (2013). Intratumor heterogeneity in human glioblastoma reflects cancer evolutionary dynamics. Proc Natl Acad Sci U S A.

[CR56] Stupp R, Mason WP, van den Bent MJ, Weller M, Fisher B, Taphoorn MJ (2005). Radiotherapy plus concomitant and adjuvant temozolomide for glioblastoma. N Engl J Med.

[CR57] Taylor K, Howard CB, Jones ML, Sedliarou I, MacDiarmid J, Brahmbhatt H (2015). Nanocell targeting using engineered bispecific antibodies. MAbs.

[CR58] Thorne RG, Nicholson C (2006). In vivo diffusion analysis with quantum dots and dextrans predicts the width of brain extracellular space. Proc Natl Acad Sci U S A.

[CR59] van Tellingen O, Yetkin-Arik B, de Gooijer MC, Wesseling P, Wurdinger T, de Vries HE (2015). Overcoming the blood-brain tumor barrier for effective glioblastoma treatment. Drug Resist Updates.

[CR60] Verhaak RG, Hoadley KA, Purdom E, Wang V, Qi Y, Wilkerson MD (2010). Integrated genomic analysis identifies clinically relevant subtypes of glioblastoma characterized by abnormalities in PDGFRA, IDH1, EGFR, and NF1. Cancer Cell.

[CR61] Vichai V, Kirtikara K (2006). Sulforhodamine B colorimetric assay for cytotoxicity screening. Nat Protoc.

[CR62] Vlachos IS, Kostoulas N, Vergoulis T, Georgakilas G, Reczko M, Maragkakis M (2012). DIANA miRPath v.2.0: investigating the combinatorial effect of microRNAs in pathways. Nucleic Acids Res..

[CR63] Vlachos IS, Paraskevopoulou MD, Karagkouni D, Georgakilas G, Vergoulis T, Kanellos I (2014). DIANA-TarBase v7.0: indexing more than half a million experimentally supported miRNA: mRNA interactions. Nucleic Acids Res..

[CR64] Vlachos IS, Zagganas K, Paraskevopoulou MD, Georgakilas G, Karagkouni D, Vergoulis T (2015). DIANA-miRPath v3.0: deciphering microRNA function with experimental support. Nucleic Acids Res..

[CR65] Wang J, Cazzato E, Ladewig E, Frattini V, Rosenbloom DI, Zairis S (2016). Clonal evolution of glioblastoma under therapy. Nat Genet.

[CR66] Wolburg H, Noell S, Fallier-Becker P, Mack AF, Wolburg-Buchholz K (2012). The disturbed blood-brain barrier in human glioblastoma. Mol Aspects Med.

[CR67] Xue W, Dahlman JE, Tammela T, Khan OF, Sood S, Dave A (2014). Small RNA combination therapy for lung cancer. Proc Natl Acad Sci.

[CR68] Yardeni T, Eckhaus M, Morris HD, Huizing M, Hoogstraten-Miller S (2011). Retro-orbital injections in mice. Lab Anim.

[CR69] Zhang J, Li S, Li L, Li M, Guo C, Yao J (2015). Exosome and exosomal microRNA: trafficking, sorting, and function. Genomics Proteomics Bioinform.

[CR70] Zhang L, Liao Y, Tang L (2019). MicroRNA-34 family: a potential tumor suppressor and therapeutic candidate in cancer. J Exp Clin Cancer Res CR.

