# Supplementary material for: Nanocell-mediated delivery of miR-34a counteracts temozolomide resistance in glioblastoma
Source: Mol Med. 2021 Mar 25;27:28. doi: 10.1186/s10020-021-00293-4 (PMC7993499; doi:10.1186/s10020-021-00293-4)
Supplement: Supplementary file 1 — Additional file 1: Table S1. List of genes predicted to be regulated by miR-34a in glioblastoma driver pathways. miRPATH v.3 was used to identify genes in the KEGG glioma pathway which are predicted to be regulated by miR-34a. miRNA-gene interactions cataloged in Tarbase v.7 were used. The cell lines, tissues of origin and the methods used to identify miR-34a gene interactions are included. IP, immunoprecipitation, RA, Reporter Gene assay, WB, Western Blot, MA, microarrays, Bi, Biotin, qP, quantitative polymerase chain reaction, CLASH, crosslinking, ligation, and sequencing of hybrids. [file 10020_2021_293_MOESM1_ESM.pdf]

Gene	Regulation	Tissue	Cell Line	Identification Methods
BRAF	Down-Regulated	Kidney	HEK293	IP
PDGFRA	Down-Regulated	Bone Marrow, Stomach	HS5, AGS	IP,RA,WB
E2F1	Down-Regulated	Intestine	HCT116	MA
CDK4	Down-Regulated	Kidney	HEK293	IP
E2F2	Down-Regulated	Intestine, Bone Marrow, Lung	HCT116, K562, A549	MA, Bi,RA,WB
MAP2K2	Down-Regulated	Intestine	HCT116	Bi
TGFA	Down-Regulated	Intestine	HCT116	Bi
PIK3R2	Down-Regulated	Cervix, Intestine	HELA,HCT116	RA,WB
RAF1	Down-Regulated	Kidney, Intestine	HEK293, HCT116	IP,Bi
IGF1R	Down-Regulated	Kidney	HEK293	CLASH
EGFR	Down-Regulated	Intestine	HCT116	Bi
CDKN2A	Down-Regulated	Intestine	HCT116	Bi
CDK6	Down-Regulated	Kidney, Liver, Bone Marrow	HEK293,7860, ACHN, HEPG2, K562	IP, WB,qP,RA
ARAF	Down-Regulated	Cervix, Intestine, Bone Marrow	HELA,HCT116, K562	RA,MA,WB,qP
TP53	Down-Regulated	Intestine, Bone Marrow	HCT116, K562	Bi
AKT2	Down-Regulated	Bone Marrow	K562	Bi
PLCG1	Down-Regulated	Intestine	HCT116	Bi
CCND1	Down-Regulated	Kindey, Bone Marrow	7860,ACHN, K562	qP,WB,Bi
E2F3	Down-Regulated	Kidney, Bone Marrow	293S,HEK293, K562	IP,qP
MAPK3	Down-Regulated	Kindey	HEK293	CLASH

PRKCB	Down-Regulated	Bone Marrow	K562	Bi
IGF1	Down-Regulated	Bone Marrow	K562	MA
PIK3CA	Down-Regulated	Bone Marrow	HS5, AGS	IP
CDKN1A	Up-Regulated	Kindey	7860,ACHN,SN12PM6	qP,WB
MAP2K1	Down-Regulated	Cervix, Intestine	HELA, HCT116	RA,qP,WB
PTEN	Down-Regulated	Bone Marrow	K562	Bi
MAPK1	Down-Regulated	Mammary Gland, Intestine	BT474, HCT116	IP,Bi
GRB2	Down-Regulated	Kidney	HEK293	IP
PDGFRB	Down-Regulated	Stomach	AGS	RA,WB

Additional file 1: Table S1
