# Supplementary material for: Nanocell-mediated delivery of miR-34a counteracts temozolomide resistance in glioblastoma
Source: Mol Med. 2021 Mar 25;27:28. doi: 10.1186/s10020-021-00293-4 (PMC7993499; doi:10.1186/s10020-021-00293-4)
Supplement: Supplementary file 2 — Additional file 2: Table S2. List of miRNAs which regulate glioblastoma driver pathways. miRPATH v 3.0 was used to explore miRNAs which regulate driver pathways in glioma as described in KEGG database. miRNA–gene interactions cataloged in Tarbase v.7 were used in this search. miRPATH v 3.0 was used to calculate enrichment p values by the one tailed Fisher’s exact test. Top thirty miRNAs from this search are listed. [file 10020_2021_293_MOESM2_ESM.pdf]

miRNA	Number of genes targeted	Enrichment p value
hsa-miR-34a-5p	29	1.88E-97
hsa-miR-17-5p	23	4.51E-74
hsa-let-7a-5p	22	2.62E-70
hsa-miR-16-5p	19	2.97E-59
hsa-miR-7-5p	19	2.97E-59
hsa-miR-424-5p	19	2.97E-59
hsa-miR-93-5p	19	2.97E-59
hsa-miR-19a-3p	19	2.97E-59
hsa-miR-27a-3p	18	1.20E-55
hsa-let-7b-5p	18	1.20E-55
hsa-miR-522-5p	17	4.38E-52
hsa-let-7g-5p	17	4.38E-52
hsa-miR-181a-5p	17	4.38E-52
hsa-miR-107	17	4.38E-52
hsa-let-7i-5p	16	1.46E-48
hsa-miR-20a-5p	16	1.46E-48
hsa-miR-497-5p	16	1.46E-48
hsa-miR-182-5p	16	1.46E-48
hsa-let-7f-5p	16	1.46E-48
hsa-miR-26b-5p	16	1.46E-48
hsa-miR-103a-3p	16	1.46E-48
hsa-miR-106b-5p	15	4.37E-45
hsa-miR-122-5p	15	4.37E-45
hsa-let-7d-5p	15	4.37E-45
hsa-miR-30a-5p	15	4.37E-45
hsa-miR-124-3p	15	4.37E-45
hsa-miR-19b-3p	15	4.37E-45
hsa-miR-92a-3p	14	1.18E-41
hsa-miR-1291	14	1.18E-41
hsa-miR-15a-5p	14	1.18E-41

Additional file 2: Table S2
