# Supplementary material for: Nanocell-mediated delivery of miR-34a counteracts temozolomide resistance in glioblastoma
Source: Mol Med. 2021 Mar 25;27:28. doi: 10.1186/s10020-021-00293-4 (PMC7993499; doi:10.1186/s10020-021-00293-4)
Supplement: Supplementary file 3 — Additional file 3: Table S3. miR-34a down-regulates multiple therapeutic resistance genes. GBM6, GBM118 and GBM126 primary cultures were transfected with 30 nM miR-34a or 30 nM miR-C. Total RNA was extracted 48 h post transfection and RT2 Profiler™ PCR Array from Qiagen was used to examine expression changes of 84 drug resistance genes. The table shows fold-regulation of genes with fold-regulation cut-off > 1.5. Fold-change was calculated by dividing the normalized gene expression (2^ (- Delta CT)) in the miR-34a transfected cells by normalized gene expression (2^ (- Delta CT)) in the miR-C transfected cells. Fold-Regulation is equal to fold-change for fold-change values > 1. For fold-change values < 1, fold-regulation is the negative inverse of fold-change. [file 10020_2021_293_MOESM3_ESM.pdf]

GBM 6		GBM 118		GBM 126	
Gene Symbol	Fold Regulation	Gene Symbol	Fold Regulation	Gene Symbol	Fold Regulation
CYP2C19	2	AR	1.72	CYP2C8	6.45
ABCC5	1.58	CYP3A5	2.08	ABCB1	1.68
SOD1	1.52	EGFR	1.85	CYP3A4	2.36
ABCB1	-6.14	EPHX1	1.71	ABCC2	-2.04
ABCG2	-120.07	MSH2	1.93	APC	-1.62
AHR	-39.61	ABCB1	-1.52	ELK1	-4.03
APC	-6.58	AHR	-2.02	ESR1	-2.45
ATM	-2.08	CDKN2D	-2.34	ESR2	-1.68
BCL2	-2.25	CLPTM1L	-2.37	NFKB1	-1.68
BLMH	-2.05	CYP2D6	-6.04	NFKBIB	-1.73
BRCA2	-2.22	CYP2E1	-2.98	NFKBIE	-2.17
CYP2D6	-2.29	ERCC3	-1.92	PPARG	-1.85
CYP2E1	-4.59	FGF2	-1.61	RARG	-1.56
CYP3A4	-5.69	GSTP1	-1.69	XPC	-1.85
EGFR	-3.03	IGF2R	-1.69		
ERBB4	-2.79	RARG	-1.6		
FGF2	-2.04	UGCG	-1.81		
HIF1A	-2.41				
MET	-2.94				
NAT2	-2.13				
PPARA	-2.37				
PPARD	-2.69				
PPARG	-8.99				
RARB	-2.69				
TPMT	-2.56				
XPA	-2.17				

Additional file 3: Table S3
