# Supplementary figures and images for: Nanocell-mediated delivery of miR-34a counteracts temozolomide resistance in glioblastoma
Source: Mol Med. 2021 Mar 25;27:28. doi: 10.1186/s10020-021-00293-4 (PMC7993499; doi:10.1186/s10020-021-00293-4)

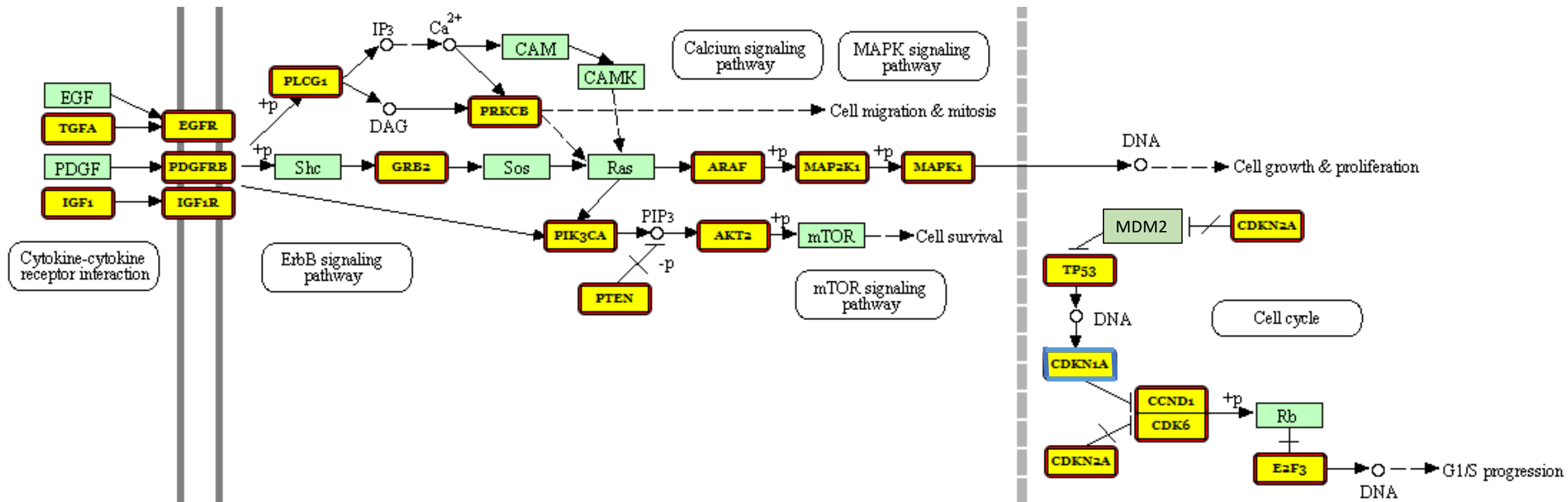

Supplement: Supplementary file 4 — Additional file 4: Figure S1. miR-34a modulates the expression of multiple signaling elements in glioma. The glioma pathway from KEGG (hsa05214) with miR-34a targets is illustrated. Yellow boxes represent genes whose expression has been reported to be modulated by miR-34a in the TarBase v 7.0. Yellow boxes with red outlines are down-regulated, while yellow boxes with blue outlines are up-regulated by miR-34a. Green boxes represent glioma signaling elements not modulated by miR-34a. [file 10020_2021_293_MOESM4_ESM.pdf]

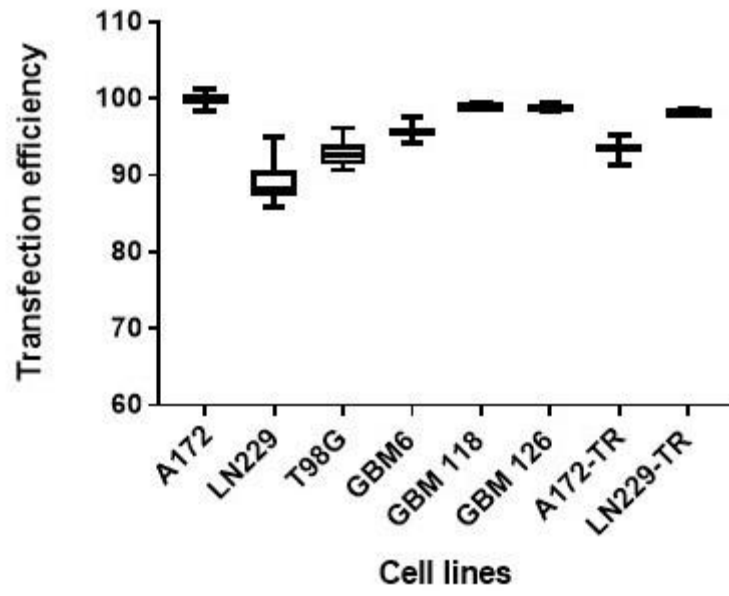

Supplement: Supplementary file 5 — Additional file 5: Figure S2. Transfection efficiency is comparable across the different cell lines tested. Cells were reverse-transfected with the TOX transfection siRNA. Successful transfection results in cell death which was quantified by SRB assay. All data represent mean SRB values (± SD) from three independent experiments. [file 10020_2021_293_MOESM5_ESM.pdf]

**A**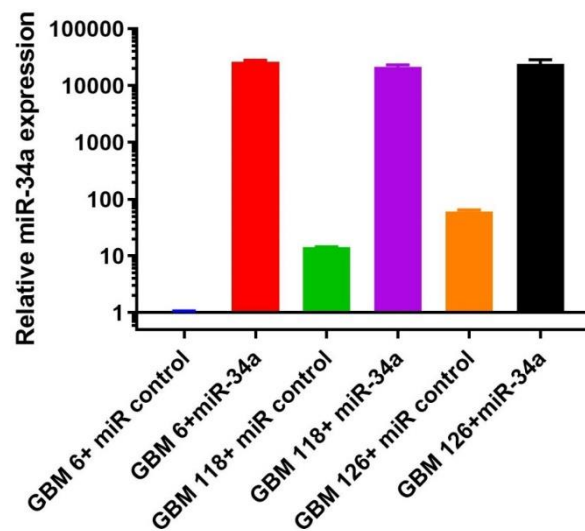**B**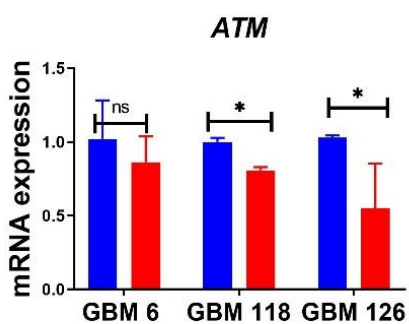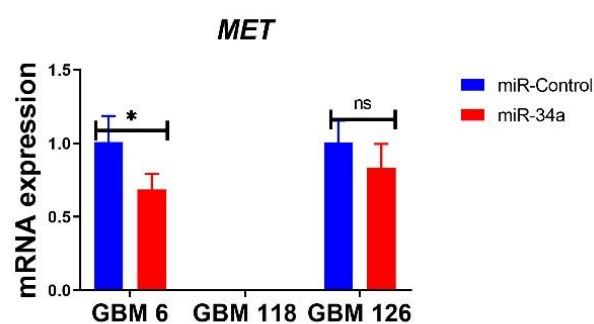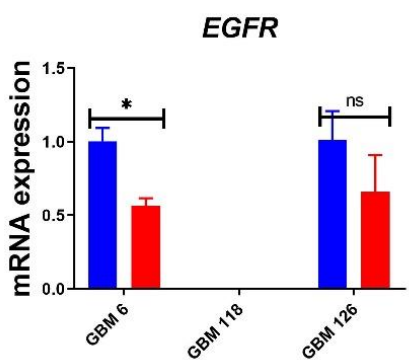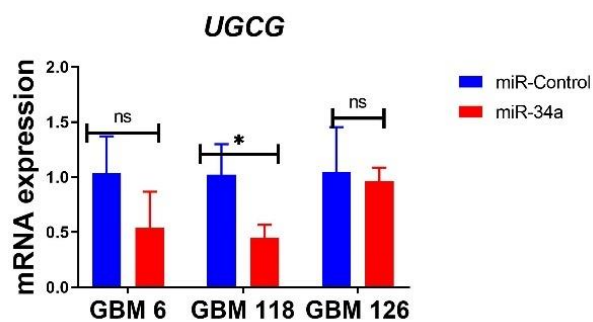

Supplement: Supplementary file 6 — Additional file 6: Figure S3. miR-34a downregulates multiple therapeutic resistance genes. A. Transfection with miR-34a significantly increases miR-34a expression in glioblastoma primary cultures. GBM6, GBM118 and GBM126 cells were transfected with 30 nM control miRNA or miR-34a. Total RNA was isolated 48 h post transfection. RNU-6 was used as housekeeping gene and relative expression calculations were made according to the Livak method. Data show mean ± SD of three technical replicates. B. miR-34a reduces to expression of multiple therapeutic resistance genes in glioblastoma. RTqPCR experiments were performed with independent primers to verify the results of the PCR array. In some instances, expression of genes could not be detected in one or more cultures by the PCR. The corresponding bars are left missing from the figure. RNU-6 was used as housekeeping gene and relative expression calculations were made according to the Livak method. Data was normalized to non-transfected controls and show mean ± SD of three technical replicates. *p < 0.05 of miR-34a cells compared to control transfected cells, n/s implies p > 0.05 [file 10020_2021_293_MOESM6_ESM.pdf]

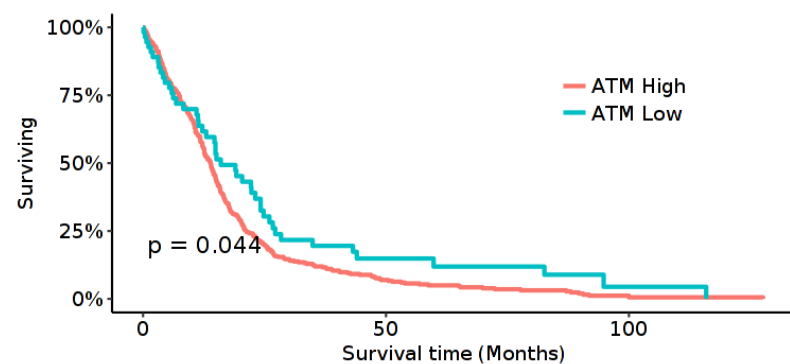

***ATM***

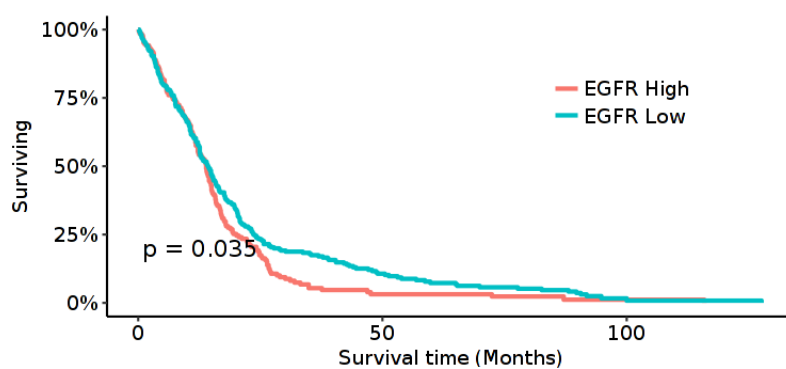

***EGFR***

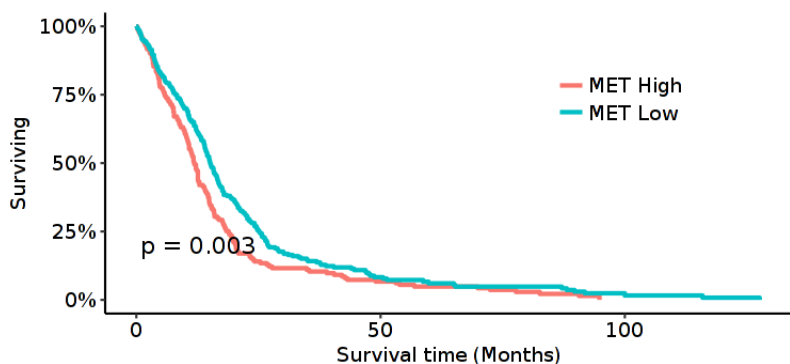

***MET***

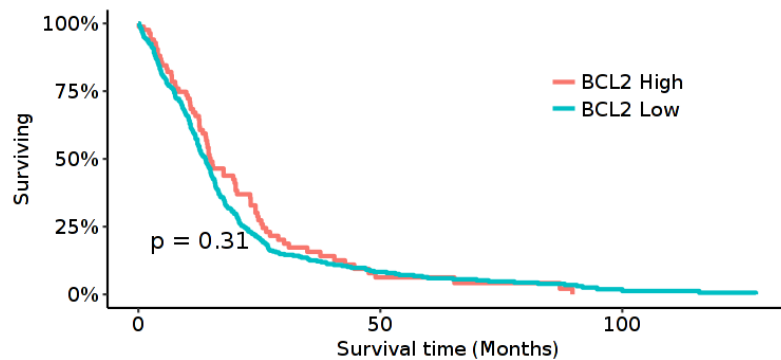

***BCL2***

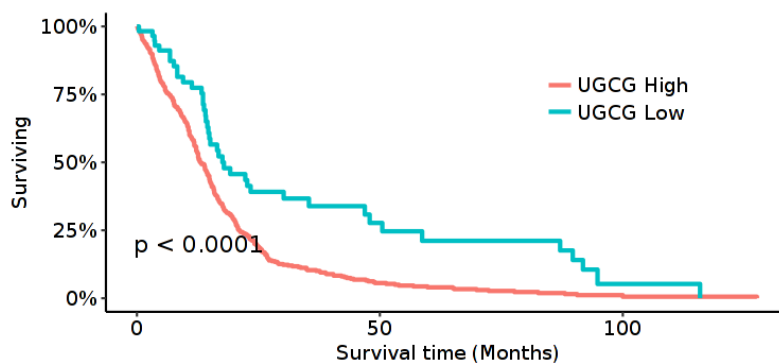

***UGCG***

Supplement: Supplementary file 7 — Additional file 7: Figure S4. High expression of miR-34a down-regulated therapeutic resistance genes is associated with worse survival. Kaplan-Meir curves for overall survival in 585 glioblastoma patients from TCGA dataset for the validated resistance genes. High expressors are plotted in orange and low expressors in blue. Cut-off mRNA expression scores used included ATM (5.47), EGFR (6.6)MET (4.37), BCL2 (3.9) and UGCG (7.8). Maximally selected rank statistic was used to stratify patients into high and low expressors. The log rank test was used to assess statistical significance. [file 10020_2021_293_MOESM7_ESM.pdf]

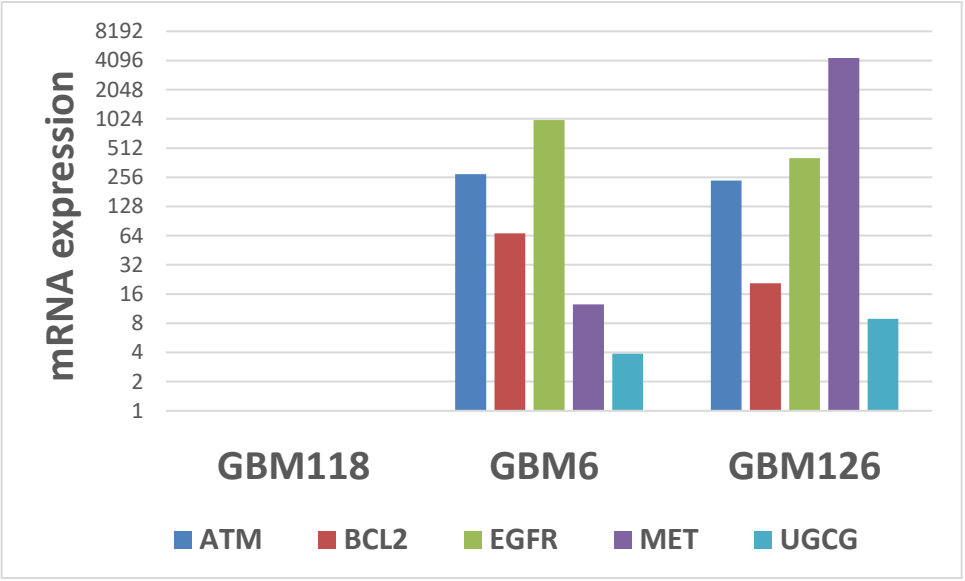

Supplement: Supplementary file 8 — Additional file 8: Figure S5. Glioblastoma cultures have different baseline expression of therapeutic resistance gens. Total RNA was extracted from GBM6, GBM118 and GBM126 primary cultures and RT2 Profiler™ PCR Array from Qiagen was used to determine baseline levels of ATM, BCL2, EGFR, MET and UGCG in these cultures. To determine relative mRNA expression, fold-change was calculated by dividing the normalized gene expression (2^ (- Delta CT)) in the GBM6 and GBM126 cells by normalized gene expression (2^ (- Delta CT)) in the GBM118 cells. [file 10020_2021_293_MOESM8_ESM.pdf]
